# Investigation of ultra-precision planing process to fabricate high luminance retroreflector based on cutting force and tool vibration analysis

**DOI:** 10.1038/s41598-022-10824-6

**Published:** 2022-05-09

**Authors:** Ji-Young Jeong, Jun Sae Han, Chung-Mo Kang, Eun-Ji Gwak, Doo-Sun Choi, Tae-Jin Je

**Affiliations:** 1 Department of Nano Mechatronics Engineering, University of Science and Technology (UST), Daejeon, 34113 Republic of Korea; 2 Department of Nano Manufacturing Technology, Korea Institute of Machinery and Materials(KIMM), Daejeon, 34103 Republic of Korea; 3 School of Mechanical Engineering, Kyungpook National University, Daegu, 41566 Republic of Korea

**Keywords:** Engineering, Optics and photonics

## Abstract

In ultra-precision planing process, the analysis of the critical depth of cut (DOC) is required to reduce the edge blunt and micro burrs produced by size effect which decreases of the effective area for high luminance retroreflector. However, since the machining characteristics are different according to cutting tool shape, machining material, and cutting condition, determine of the critical DOC is difficult without a comparison of machined surfaces under various DOC measured by ultra-high resolution measuring instrument. In this study, the critical DOC was analyzed using cutting force and tool vibration signals. The specific cutting energy was calculated by cutting force and cross-sectional area to analyze the stress variation according to DOC. Also, acceleration signals were converted to frequency spectrum that analyze dominant vibrating direction of the cutting tool by variation of cutting characteristic. It was confirmed that the method of using tool vibration more effective and accurate than specific cutting energy through validation of the comparison between results from analyze of the vibration signals and direction measuring surfaces. The master mold with area of 250 mm^2^ was manufactured by applying analyzed critical DOC. In addition, the high luminance characteristic of a retroreflection film press formed by the master mold was confirmed.

## Introduction

The surfaces with micro-structures can change the optical path of the light propagation. The controlled light propagation can achieve the optical performance such as retroreflection, light diffusion, concentration, etc.^1–5^. In these optical characteristics, optical retroreflection is widely used in various industries such as night safety signs, traffic signs, vehicle rear reflectors, display, solar-cell, and advanced optical measuring devices^6–11^. The main parameter of the retroreflection performance is the brightness of the retroreflected light, it was quantified as the ratio of the reflected light intensity to the incident light. Ultra-high brightness retroreflectors can be fabricated by using micro-triangular pyramid array that returns incident lights by total reflection from three surface of inside the pattern, and it is required to minimize the light scattering by the surface defects for higher efficient retroreflection.

The manufacturing technologies for fabricating micro-triangular pyramid patterns are including chemical etching process^12,13^, laser machining process^14,15^, and lithography process^16^. Even though these technologies enable to fabricate the various shape and fine structure for the retroreflection, but fabricated patterns typically have a blunt shape and a rough surface. The blunt edges can reduce efficiency of the total reflection of the incident light by reduced area for reflection, and the rough surface can scatter the light on the incident surface. For these reasons, these problems can deteriorate efficiency of the retroreflection.

**Figure 1:**
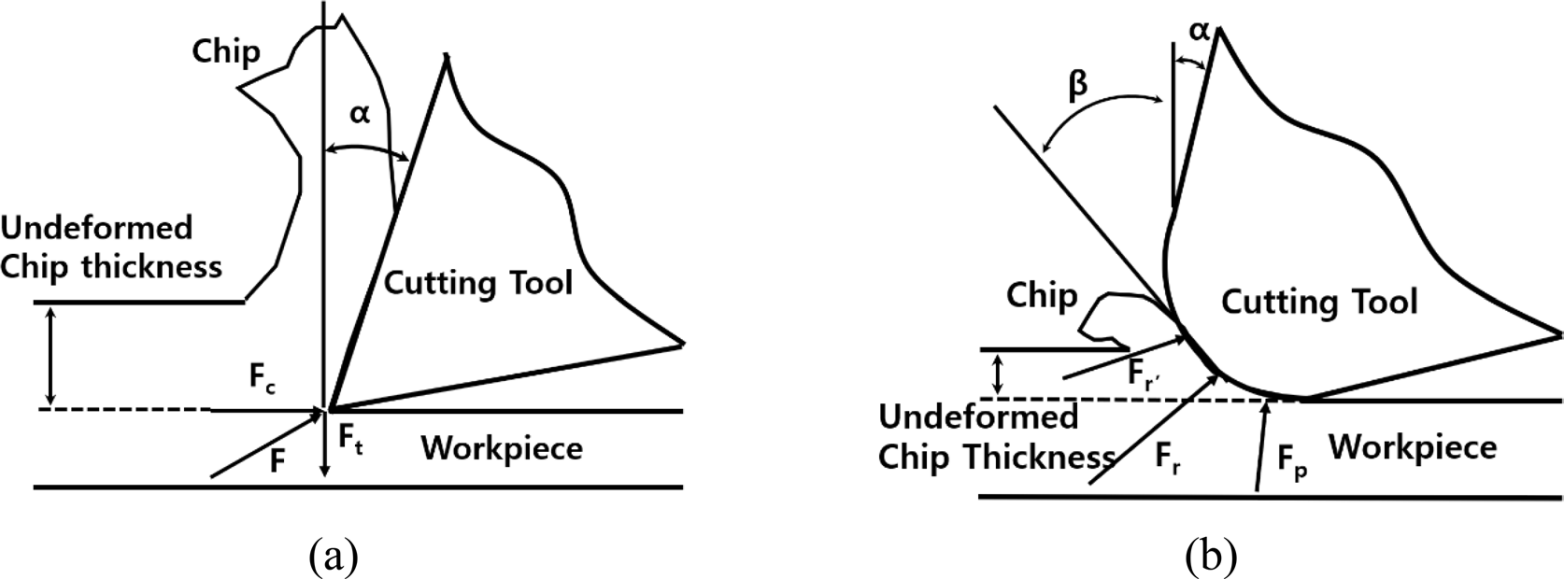
Schematic of two-dimensional steady state orthogonal cutting process (**a**) undeformed chip thickness bigger than radius of cutting tool edge (**b**) undeformed chip thickness smaller than radius of cutting tool edge.

The ultra-precision cutting process using a diamond cutting tool have advantages which can fabricate smooth surface with roughness value less than 10 nm level and shape with sharp edges^17,18^. Typically, the cutting process known to fabricate the high-quality of the machined surface applying cutting conditions which is shallow depth of cut and fast cutting speed^19,20^. However, there is a critical DOC that was difficult to ignore the force acting on the cutting edge due to the curvature of the cutting tool edge. Figure 1 shows the schematic of the two-dimensional steady state of the orthogonal cutting process. The resultant tool force (*F*_*r*_) in metal cutting is distributed over the contact area between tool and workpiece. When a DOC is larger than cutting edge radius, the cutting force has small proportion of the resultant force at large value of undeformed chip thickness as shown in Fig. 1a. In this case, the resultant force can be divided to two components of cutting force (*F*_*c*_) and thrust force (*Ft*). However, at the small value of undeformed chip thickness as shown in Fig. 1b, the cutting tool edges plows the work material during the cutting process because of inevitably generated edge radius by joining surfaces which are flank and rake face. In this case, because the force acting on the cutting edge cannot be neglected, the resultant force can be presented by sum of two component force which are plowing force (*F*_*p*_) and shearing force (*F*_*r’*_). The plowing force is act on the flank surface of the cutting tool that do not contribute to removal of the chip, and it generate the important phenomenon so-called size effect^20^. The cause of the size effect is presented by Nakayama, Kazuo and Tamura, Kiyoshi in research of cutting force according to DOC. As a resultant of their study, the main cause of the size effect is the decreasing of shear angle with a decreasing the DOC due to the blunt shape of cutting edge^25^. Because the machined surface has problems such as burr and deformation when a size effect is acting in cutting process, determination and analyzation of the critical DOC which generate the size effect is important^20–22^. Liu, H. and Xu, H. are studied to decrease the burrs generated on the edge of micro pyramid pattern using single-edge cutting method^23,24^. The chip flowing was simulated and compared with experimentally machined results according to cutting methods. Even though this study was obtained superior results that the ratio of optical functional area can increase 10.1% by reducing the size of burrs, the Possin burr due to the size effect still exists, and the analysis technology of critical DOC is required to minimize the burr formation. Specific cutting energy is widely applied^21,22^ to analyze the critical DOC. However, the specific cutting energy is calculated as the ratio of the cutting force in the cutting direction and cutting area, it is insufficient to accurately analyze the critical DOC with size effect by plowing force acting on the cutting edge in various direction. In other hands, because the cutting tool vibration has the various information of the characteristics in cutting process, method of analyzing tool vibration can be efficiently applied to determine the critical DOC.

In this study, the specific cutting energy and vibration of the cutting tool were analyzed according to the DOC in the finish machining for Cu electroplated metal mold with micro triangular pyramid pattern array. The critical DOC was determined by the results from variation of the specific cutting energy and the frequency of tool vibration. An experimental study was carried out for validity of using tool vibration signal to determine the critical DOC. Then, it was adapted to fabrication process as a finish cutting condition, the large area master mold with superior quality of surface and edge was manufactured, and the retroreflection performance of molded film fabricated by using master mold was evaluated.

## Experiments

### Experimental set-up

**Figure 2:**
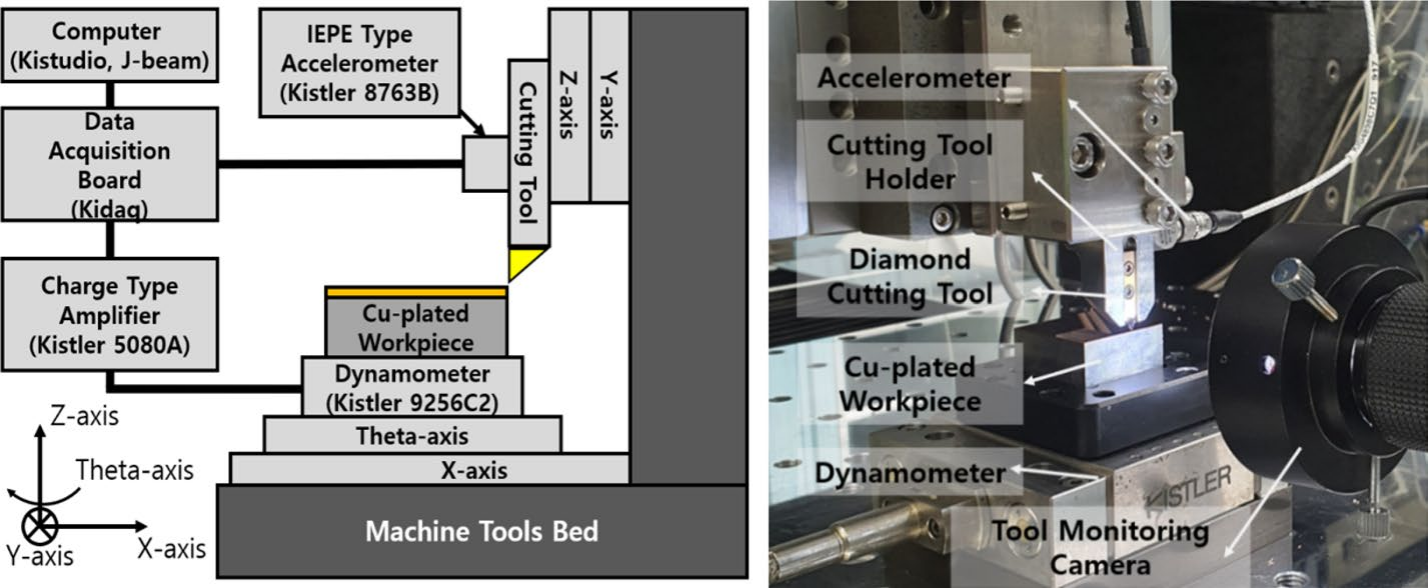
Schematic diagram of cutting force and cutting tool vibration measurement system and set-up of ultra-precision machining system for fabricating triangular pyramid pattern.

**Figure 3:**
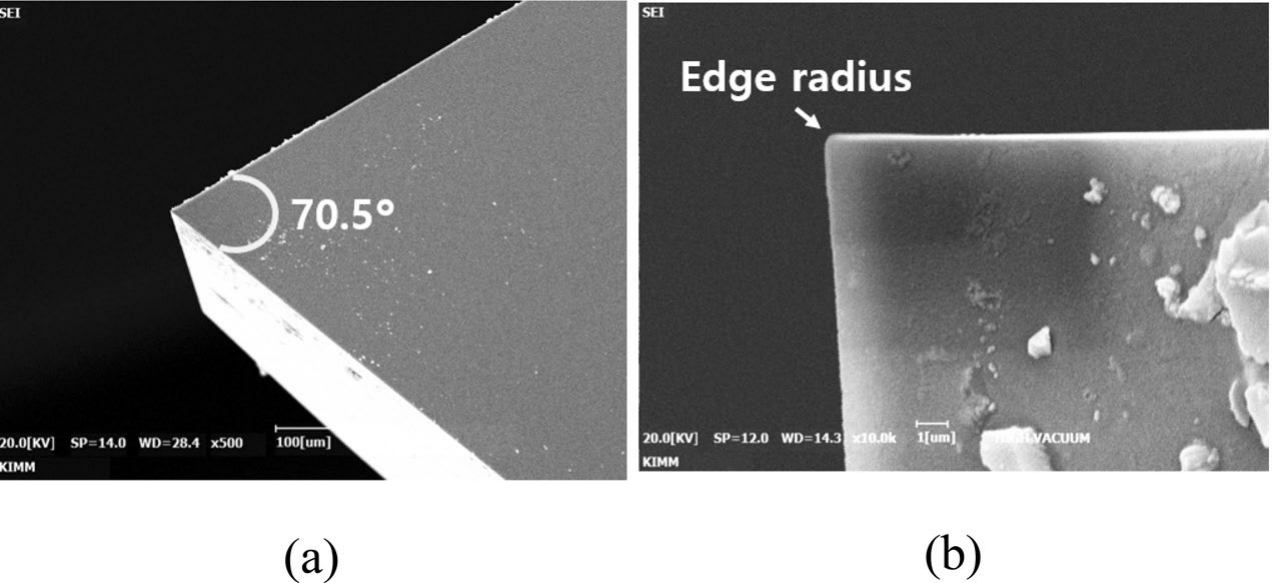
Single crystal diamond cutting tool which has sharp edge with angle of 70.5° and edge radius of 500 nm; (**a**) top view (**b**) side view.

In order to determine the critical depth of cut which is generate the size effect in machining of the micro triangular pyramid patterns, the study for analyzing cutting force and acceleration signals according to depth of cut is conducted. Figure 2 shows the schematic of experimental set-up for the collecting cutting signals of cutting force and vibration, and Fig. 3 shows the experimental system for fabricating triangular pyramid pattern on the Cu-plated mold. The machine tool was consisted of the three linear stages (X, Y, and Z-axis) with positioning resolution of 5 nm and rotation axis(theta-axis) with a resolution of 0.00001˚. For measuring cutting force, the 3-axis dynamometer (9256C2, Kistler) has sensitivity -26p C/N with direction of F_x_ and F_z_, and −13p C/N with direction of F_y_. The analog signal generated by dynamometer is converted to voltage signal with range of ± 10 V by charge amplifier (5080a, Kistler), and it was transferred to data acquisition board (DAQ, Kidaq, Kistler). In order to measure the tool vibration, an accelerometer sensor (8763B, Kistler) of integrated electronics piezo-electric (IEPE) type was attached to the shank of cutting tool. An accelerometer has sensitivity of 100 mV/g and resonance frequency of 35 kHz. The sampling rate was applied to 10 ks/s for collecting enough data, and high-frequency pass filter of 10 Hz was applied for filtering the noise signals of machine tools. The collected signals were analyzed by using software J-beam (Kistler) which is signal analyzer program.

**Figure 4:**
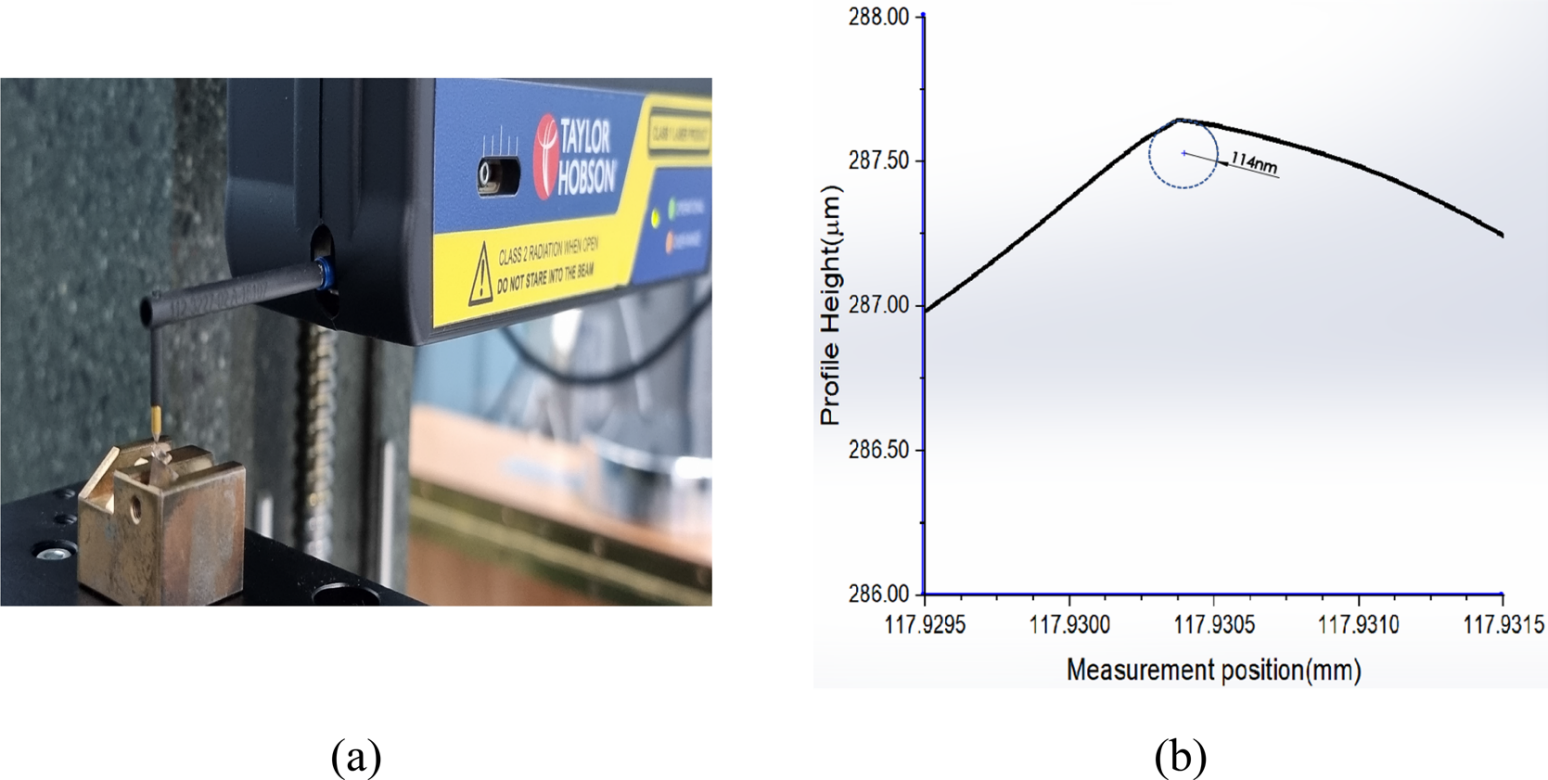
Measurement set-up and measured profile result; (**a**) Set-up for measuring cutting edge radius using high-performance profilometer (**b**) measured profile with edge radius of 114 nm.

In the planning process, because the edge radius of cutting tool is affected to generate the size effect, the measurement of the edge radius is important. Figure 3 shows the tow view and side view images of single crystal diamond(SCD) which has sharp edge with shape angle of 70.5˚. In order to measuring the cutting edge radius, the high performance profilometer(PGI-1240,TAYLOR HOBSON) with height resolution of 0.8 nm was used as shown in Fig. 4a. The stylus tip of the profilometer with a radius of 2 μm was set as precisely as possible to cross the end of diamond cutting tool. Figure 4b shows the measured profile of the diamond cutting tool. The circle was fitted by three-point draw method to measure a cutting edge radius, and it has a radius of 114 nm.

### Experimental conditions

**Figure 5:**
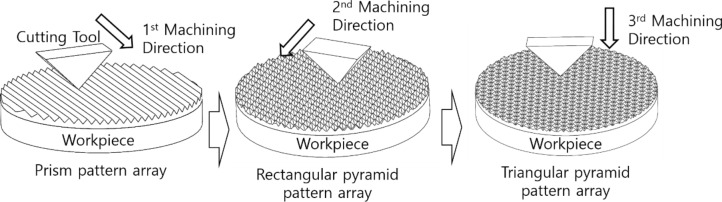
Schematic of cutting process triangular pyramid pattern.

Figure 5 shows the schematic of the planing process to fabricate a triangular pyramid pattern. This process fabricates triangular pyramid patterns by cross-machining the center of the square pyramid pattern which was fabricated by prior machining paths of -60˚ and 60˚.

**Figure 6:**
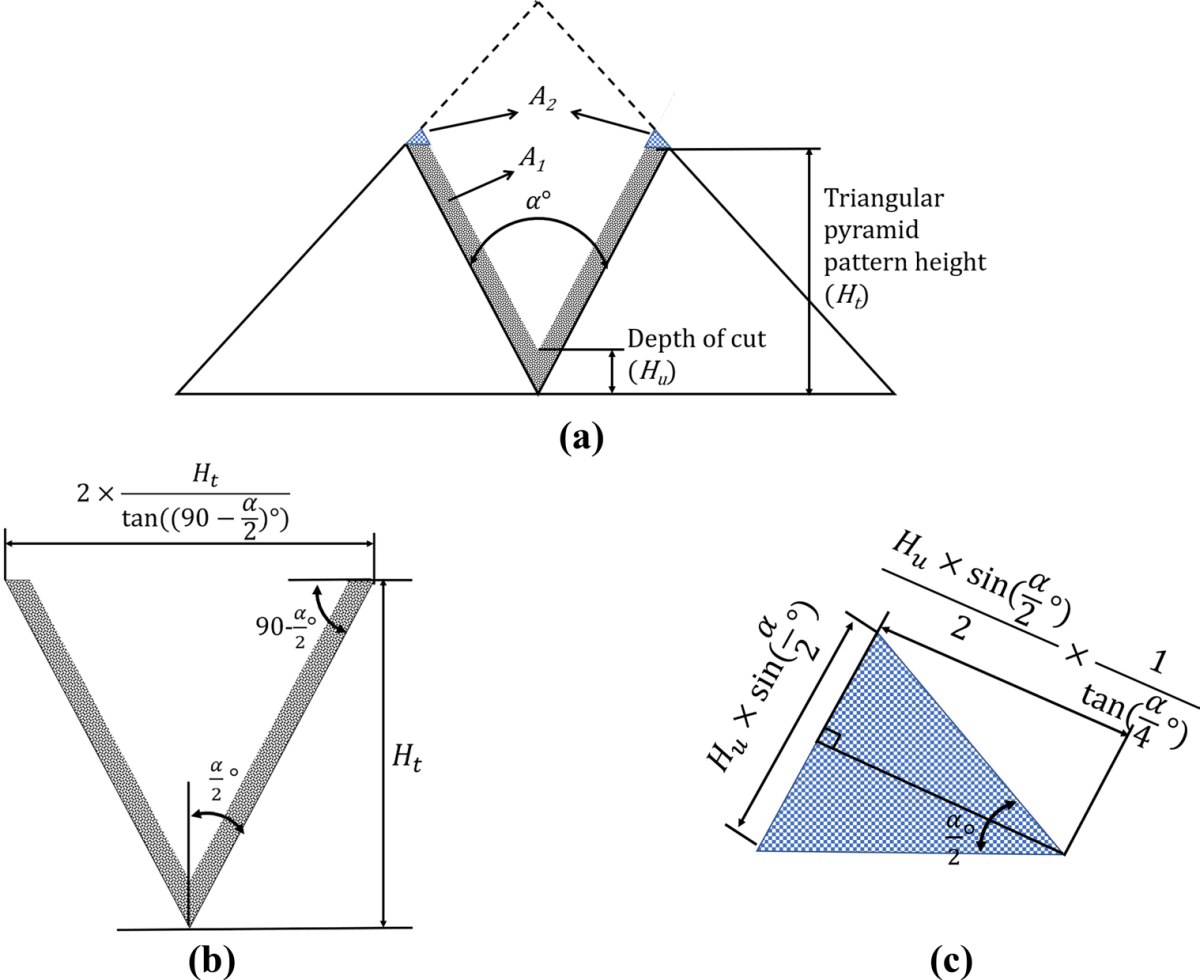
Schematic of the cutting area in the finish machining of triangular pyramid pattern by ultra-precision planning process; (**a**) total cutting area, (**b**) cutting area of *A*_*1*_ in Fig. 6. (**a**), (**c**) cutting area of *A*_*2*_ in Fig. 6 (**b**).

Figure 6 shows the cutting area of finishing in planing process of a triangular pyramid pattern with height of *H*_*t*_. Total cutting area(*A*) of Fig. 6a consists of V shaped area(*A*_*1*_) and two triangles(*A*_*2*_) as shown in Fig. 6b and c, respectively. Table 1 summarizes the values of cutting areas calculated by Eq. (1) according to depth of cut(*H*_*u*_). From these calculated values, the cutting area *A*_*2*_ does not significantly affect the size of total cutting area*.*

1 $$A = A_{1} + 2 \times A_{2} = \frac{{H_{t}^{2} - \left( {H_{t} - H_{u} } \right)^{2} }}{{{\text{tan}}\left( {90 - \frac{\alpha }{2}} \right)}} + \frac{{\left( {H_{u} \times {\text{sin}}\left( {\frac{\alpha }{2}} \right)} \right)^{2} }}{{2 \times {\text{tan}}\left( {\frac{\alpha }{4}} \right)}}$$

**Table 1:** Cutting area according to depth of cut in finishing process to fabricate triangular pyramid pattern with height of 71.425 μm using cutting tool with sharp edge of 70.5°.

Depth of cut (μm)	Cutting area *A*_*1*_ (μm^2^)	Cutting area *A*_*2*_ (μm^2^)	Total cutting area *A* (μm^2^)
3	297	2.36	299.36
2	199	1.05	200.05
1	100	0.26	100.26
0.5	50.3	0.17	50.47
0.3	32.3	0.02	32.32
0.1	10.1	0.01	10.11

Table 2 summarizes the machining conditions applied to experiments for analyzing size effect according to the variation of the DOC. The depth of cut was applied to 3, 2, 1, 0.5, 0.3, and 0.1 µm in the final machining of triangular pyramid pattern, and the total depth of this machining was 107.5 µm. The cutting speed is main factor of the machinability in cutting process. The effect on the machined quality according to cutting speed was confirmed by priorly conducted study^26^. In this prior study, the machinability on the surface and edges was improved when the cutting speed was increased from 100 to 200 mm/sec. Therefore, the cutting speed was chosen at 200 mm/sec for machining triangular pyramid structure, and cutting speed was fixed to obtain the effect on machining quality according to variation of depth of cut.

**Table 2:** Cutting conditions for analyzing the cutting signals according to depth of cut when cutting depth was 71.425 μm in machining of micro triangular pyramid pattern.

Machine tools	4-axis ultra-precision planar of X, Y, Z and theta-axis (Movement resolution of 5 nm and rotation resolution of 0.0001°)
Cutting tool	Single crystal diamond with sharp edge of 70.5° (rake angle of 0° and cutting edge radius of 114 nm)
Workpiece	Cu-plated on the SKD 11 with size of 30 × 30 mm (Vickers Hardness 227.8)
Cutting speed (mm/sec)	200
Depth of cut (µm)	3, 2, 1, 0.5, 0.3, 0.1

## Results and discussions

### Cutting force vibration according to depth of cut

**Figure 7:**
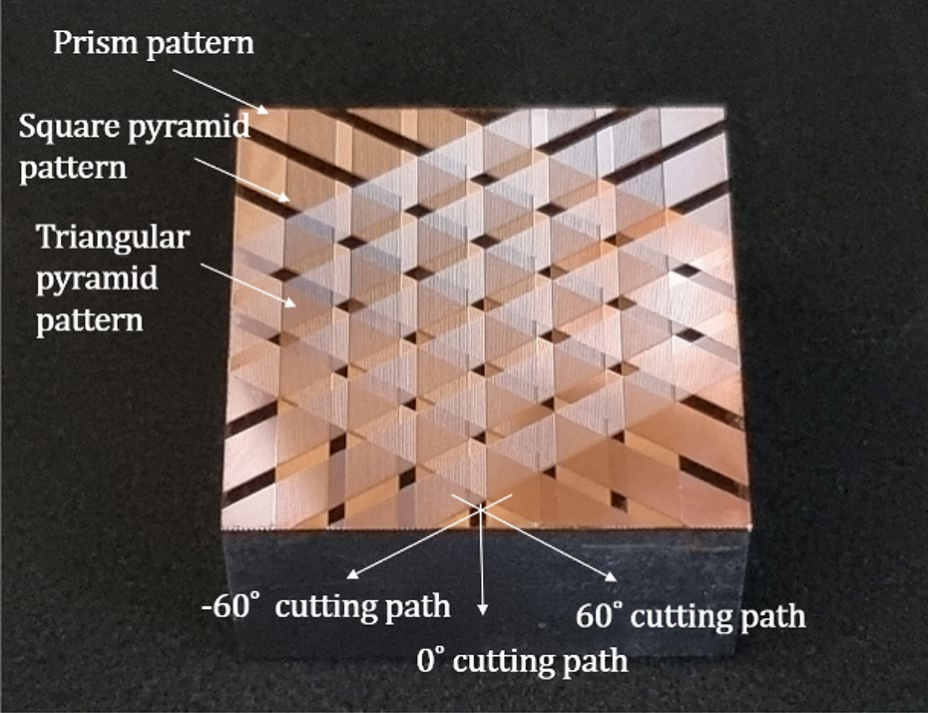
Machined workpiece by ultra-precision planing process with -60°, 0°, and 60° tool paths.

Figure 7 shows the machined workpiece, which includes the planar surface, prism pattern, square pyramid, and triangular pyramid pattern. These were generated by the cross number of the cutting tool paths which were −60°, 0°, and 60°. The prism patterns were machined by applied only one of the −60°, 0°, and 60° cutting paths. The square pyramid patterns were machined by cross machining with two cutting paths among them, and triangular pyramid patterns were machined by the cross of three cutting paths.

**Figure 8:**
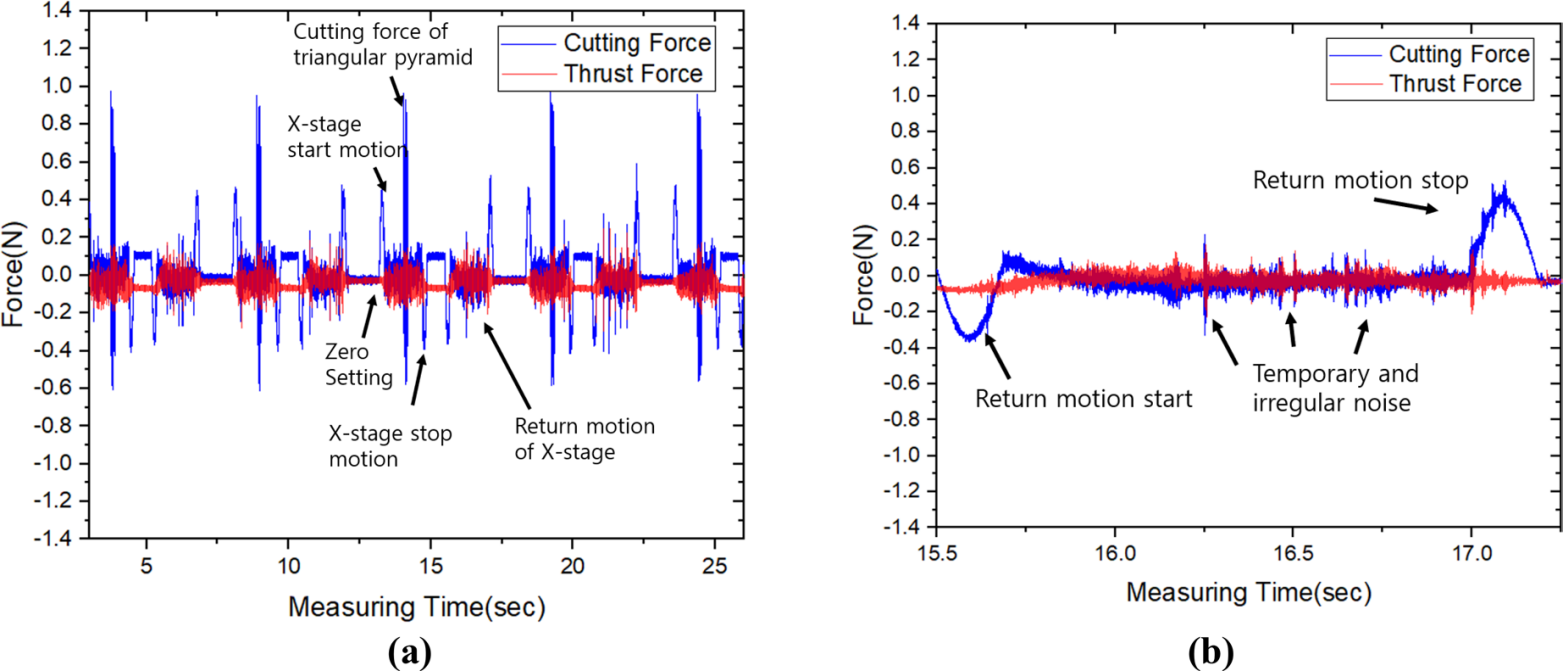
The cutting force and thrust force during five machining passes with depth of cut of 3 µm and enlarged signal at without cutting; (**a**) whole signal data, (**b**) enlarged signal in state of return motion.

The force signals were acquired during five passes of cutting process at each depth of cut. For each depth of cut, the cutting signals were measured in x-direction and z-direction. The force of x-direction was cutting force, and the force of z-direction was thrust force. and Fig. 8a representatively shows the acquired cutting force and thrust force signal at depth of cut of 3 µm. The cutting force was drastically rise and decrease to 0.4 N and −0.6 N at the point of start and stop motion because the signal communication cable was loaded by acceleration and deceleration. However, after the moving speed reach at the cutting speed at 200 mm/sec, the cutting signals were stabilized. The force signal acquired without cutting can be confirmed of noise signal by vibration. Figure 8b shows the enlarge at return motion of x-stage without cutting. In this region, the cutting force has 0.04 N RMS and thrust force has 0.02 N RMS. Even though acquired force signals has irregular noises, the cutting characteristics considered to be able analyzed because the degree of the noise difficult to affect the trend of variation of cutting force in machining of triangular pyramid pattern.

**Figure 9:**
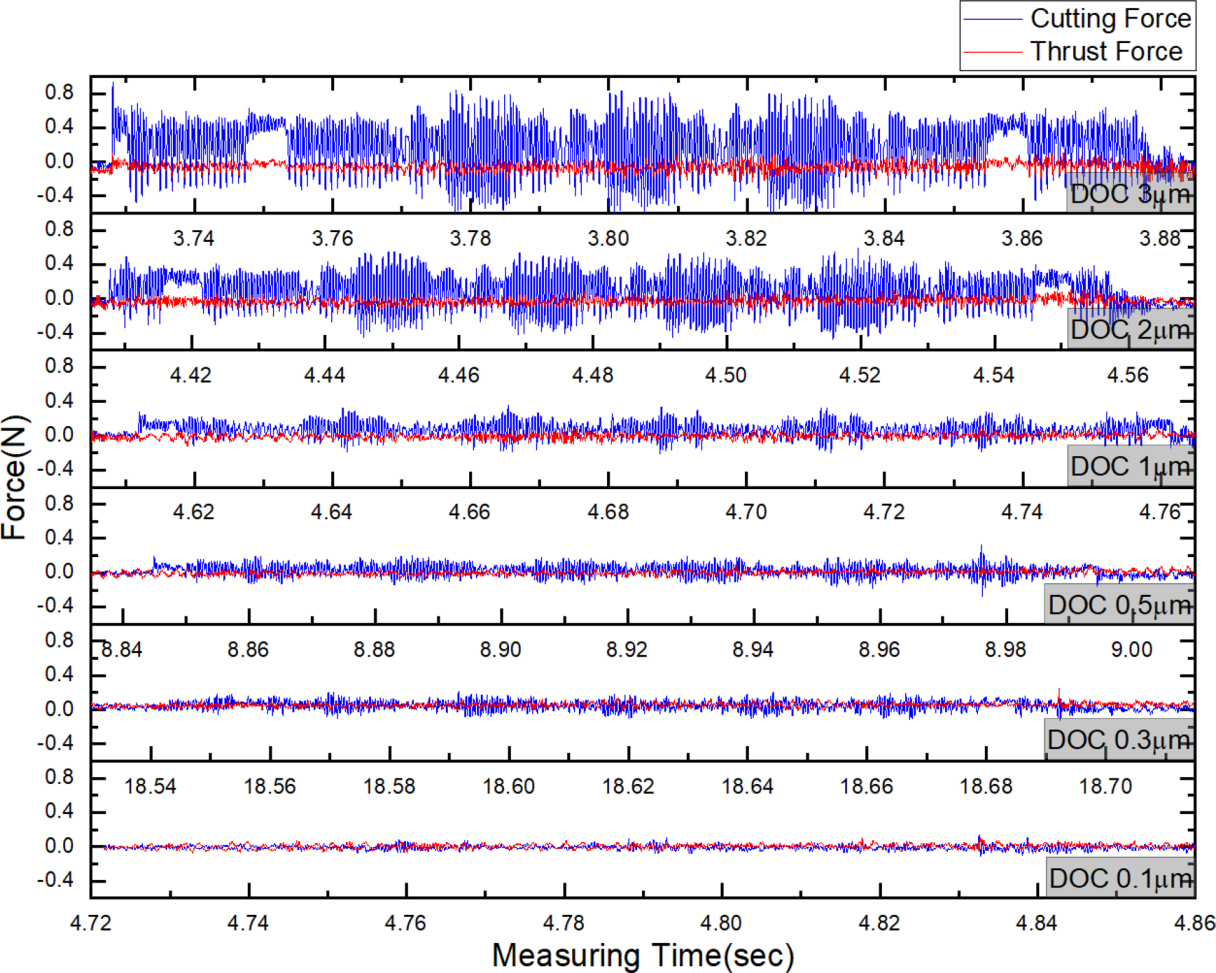
Cutting force and thrust force according to depth of cut from 3 µm to 0.1 µm at finish machining of ultra-precision planing process to fabricate miro-triangular pyramid pattern(blue line shown a cutting force and red line shown a thrust force).

Figure 9 shows the measured force signals within one pass through workpiece according to depth of cut from 3 to 0.1 µm. The measuring time of the force signals in the cutting state is about 0.152 s, it was a similar time to the calculated time of 0.15 s that the cutting tool was passed through a workpiece length of 30 mm at a feed rate of 200 mm/sec. At each cutting condition of depth of cut, the measured force signals included the machining state of prism, square pyramids, and triangular pyramids. Among these, the signal with a large amplitude is the force measured during the machining of a triangular pyramid. The cause is that the largest area was machined because the cutting tool crosses the centre of the previously machined square pyramid, and the vibration was generated by the collision between the cutting tool and workpiece due to interrupted cutting.

Typically, since the cutting force is proportional to the cutting area, the value and amplitude of the cutting force were decreasing according to shallowing DOC. The thrust force was related to the value of the consuming total cutting energy, it tended to decline as the DOC shallowing from 3 µm to 1 µm. However, with DOC lower than 500 nm, the quantitative comparison of the measured thrust force is difficult to compare directly because the signal had a large fluctuation by intermittent cutting than increment of force by material cutting. Therefore, in order to quantitative analysis, The calculation of root mean square(RMS) as the Eq. (2) was used to acquire the effective force value from the measured signals^27^.

2 $$F_{rms} = \sqrt {\frac{1}{n}\left( {F_{1}^{2} + F_{2}^{2} + F_{3}^{2} + \cdots + F_{n - 2}^{2} + F_{n - 1}^{2} + F_{n}^{2} } \right)}$$

*F*_*rms*_ is the RMS value of the measured cutting force. *F*_*n*_ is the measured force value, and n is the total number of measured force values in cutting time of the triangular pyramid pattern.

**Figure 10:**
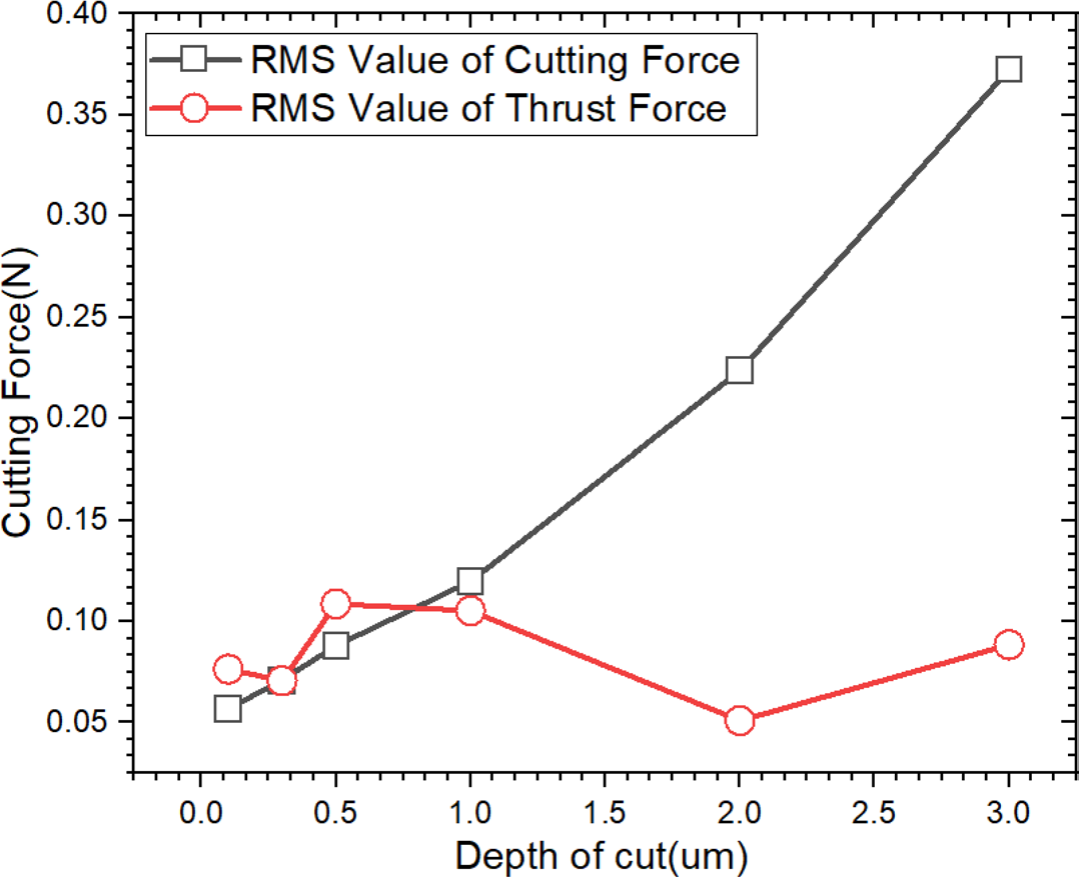
RMS value of cutting force and thrust force according to undeformed chip thickness in finish machining of triangular pyramid pattern.

Figure 10 shows the RMS values of the cutting force and thrust force according to the DOC. The RMS value of cutting force was linearly decreased from 0.3726 to 0.0567 N according to the DOC from 3 to 0.1 µm. The thrust force shows the minimum value of 0.0509 N at DOC of 2 µm, but it was increased and maintained larger than the cutting force at less than the DOC of 1 µm. This trend can be considered by plowing and deformation at flank surface of cutting tool, and it can affect the degree of thickness difference between undeformed chip thickness and machined chip thickness.

Based on a Merchant’s model, the shear angle(${\text{\O }}$) can be calculated by using cutting force and thrust force expressed as Eq. (3), and the shear plane length(${l}_{s}$) was determined as shown in Eq. (4). The $\gamma$ is working normal rake angle that is same with rake angle of cutting tool used in this study. Then, the machined chip thickness(*H*_*c*_) can be calculated by Eqs. (3) and (4) as Eq. (5). Table 3 shows the calculated values of ${\text{\O }}$, ${l}_{s}$, *H*_*c*_ and deformation ratio of chip according to undeformed chip thickness(*H*_*u*_); In this study, undeformed chip thickness is same value with DOC and. The increment of the deformation ratio of the chip thickness mean to widen the plastic deformation area of shear zone, and it also means that the energy consumption for material removal is increased. When the undeformed chip thickness reached at 1 μm the deformation ratio of the chip thickness was significantly increased more than 1 μm, and this deformation showed about double at undeformed chip thickness of 100 nm.

3 $${\text{\O }} = { }\frac{\pi }{4} - \frac{1}{2}tan^{ - 1} \left( {\frac{{F_{t} }}{{F_{c} }}} \right)$$

4 $$l_{s} = \frac{{H_{u} }}{{{\text{sin}}\emptyset }} = \frac{{H_{c} }}{{{\text{cos}}\left( {\emptyset - \gamma \left( {\frac{\pi }{180}} \right)} \right)}}$$

5 $$H_{c} = \frac{{H_{u} {\text{cos}}\left( {\frac{\pi }{4} - \frac{1}{2}tan^{ - 1} \left( {\frac{{F_{t} }}{{F_{c} }}} \right) - \gamma \left( {\frac{\pi }{180}} \right)} \right)}}{{sin\left( {\frac{\pi }{4} - \frac{1}{2}tan^{ - 1} \left( {\frac{{F_{t} }}{{F_{c} }}} \right)} \right)}}$$

The specific cutting energy ($u$) is the energy consumed per unit volume of material removal that is expressed by value of cutting energy(*U*) per material removal volume as shown in Eq. (6). The cutting energy consumption can be expressed as the product of cutting force(*F*_*c*_*)* and cutting speed(*V*_*c*_*)*, and material removal volume can be expressed as product of *V*_*c*_ and cross-sectional area of undeformed chip(*A*). Therefore, the $u$ which is calculated by of *F*_*c*_ per *A* can be considered to resistance value by stress in during cutting process for material removal that has same physical dimensions with specific cutting pressure(*K*_*s*_).

6 $$u = \frac{U}{{V_{c} \times b \times H_{u} }} = \frac{{F_{c} \times V_{c} }}{{V_{c} \times A}} = \frac{{F_{c} }}{A}$$

**Table 3:** The calculation results of shear angle, shear plane length, machined chip thickness and deformation ratio according to undeformed chip thickness based on Merchant’s model.

Undeformed chip thickness (= Depth of cut, μm)	Shear angle (°)	Shear plan length (μm)	Cutting chip thickness (μm)	Deformation ratio ((*H*_*c*_-*H*_*u*_)/*H*_*u*_)
3	38.319	4.838	3.634	0.211
2	38.588	3.206	2.398	0.199
1	24.356	2.425	2.154	1.153
0.5	19.493	1.498	1.384	1.769
0.3	22.561	0.782	0.705	1.351
0.1	18.290	0.319	0.297	1.969

**Figure 11:**
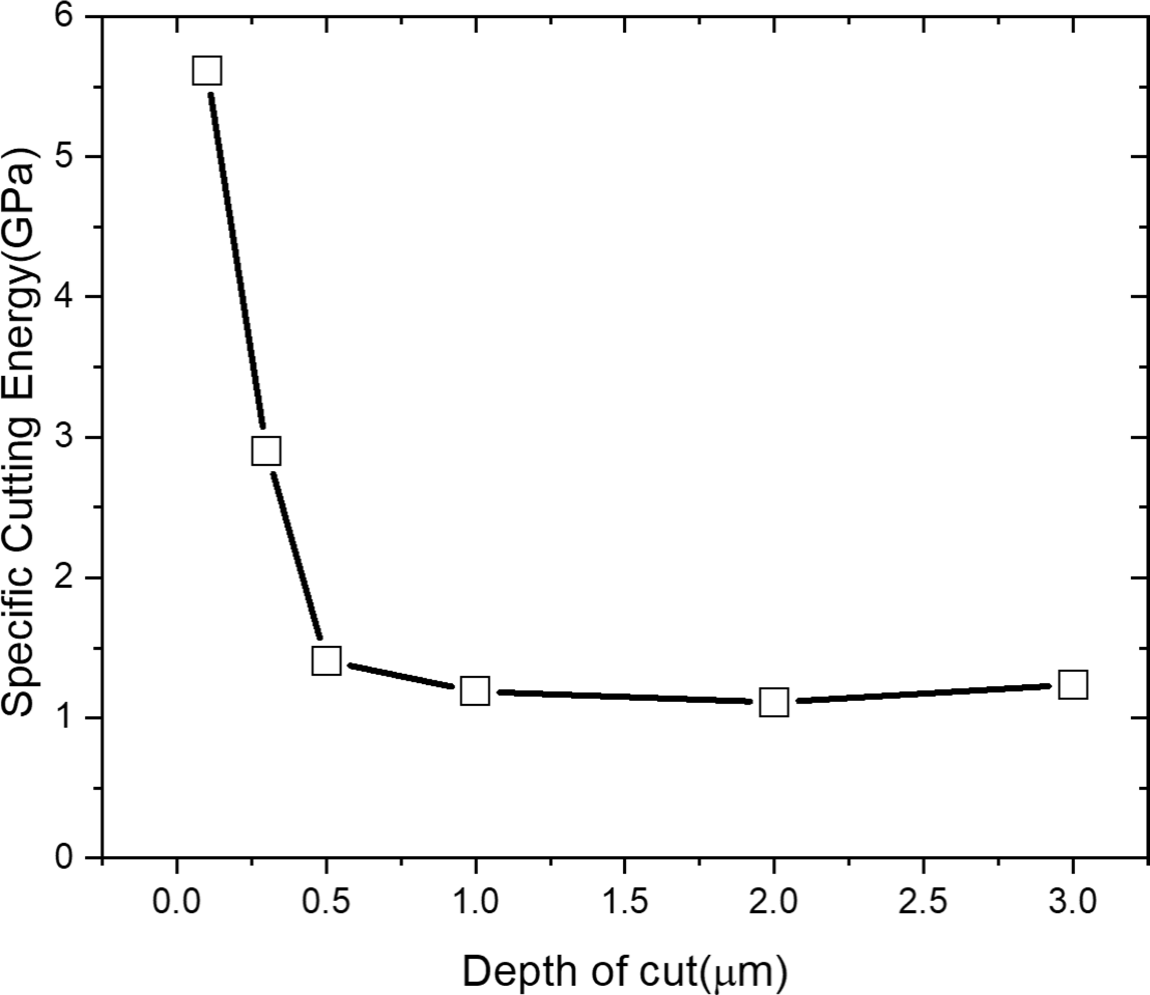
Specific cutting energy according to variation of the depth of cut.

Figure 11 shows the variation of the $u$ and cutting area according to undeformed chip thickness. The $u$ slightly declines from 1.256GPa to 1.194GPa according to undeformed chip thickness thinning from 3 to 2 µm. However, after a slight increase in the $u$ up to 1.41GPa at the DOC of 1 µm, it tended to increase significantly to 5.61GPa at 0.1 µm, which was about 4 times as much as $u$ at the DOC of 3 µm. Even though the cutting area was decreased, the cause of the increase in the $u$ was analyzed as follows. The rake angle of the cutting tool was significantly decreased where the depth of cut was lower than the edge radius. In this state, the deformation of plastic zone of shear and friction between material and cutting tool has a greater effect on the cutting process by increased stress, because the plowing force was concentrated on the edge of the cutting tool^28^. The concentrated stress was showed the increase of the $u$, and it can be estimated as a phenomenon due to the size effect. Even though the $u$ showed an increasing tendency, there is a large gap of the variation at the DOC of 1 µm between the increment of $u$ and deformation ratio of cutting chip thickness, because the Eq. (6) is simplified by assuming two-dimensional orthogonal cutting. Even though the stress value can be calculated more precisely under three-dimensional cutting process, it was difficult to many complex factors such as tool shape, angle, chip flow, etc. For this reason, the analysis method of cutting tool vibration was applied to analyze the machining characteristics using the vibration signal according to directions in parallel (X-direction in machine tools) and perpendicular direction (Z-direction in machine tools) with cutting direction.

### Cutting vibrations according to depth of cut

**Figure 12:**
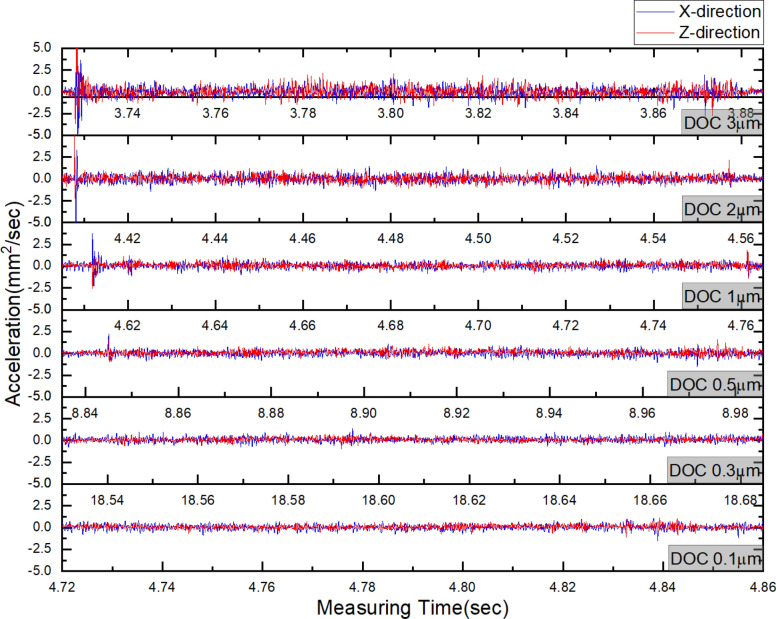
Cutting tool vibration in X-direction and Z-direction according to depth of cut(blue line shown signal in x-direction and red line shown signal in Z-direction).

Figure 12 shows the results obtained by using the acceleration sensor for measuring the vibration signal of cutting tool according to the variation of undeformed chip thickness. The acceleration of X-direction is the vibration in the cutting direction, and the acceleration in the Z-direction is the vibration of perpendicular direction to the cutting direction. The measured accelerations in the X and Z directions showed similar amplitudes within the respective machining conditions. The maximum value of the acceleration within cutting state was decreased from 2.252 to 1.12 m/sec^2^ according to decrease the undeformed chip thickness. Even though this result can indicate the size variation of tool vibration, but it was difficult to indicate the cutting characteristic that was varied by size effect. However, the vibration signal includes many information about characteristic of cutting process, and each information can be confirmed by the frequency domain signal which was converted from time domain signal by using Fourier transform^29^.

**Figure 13:**
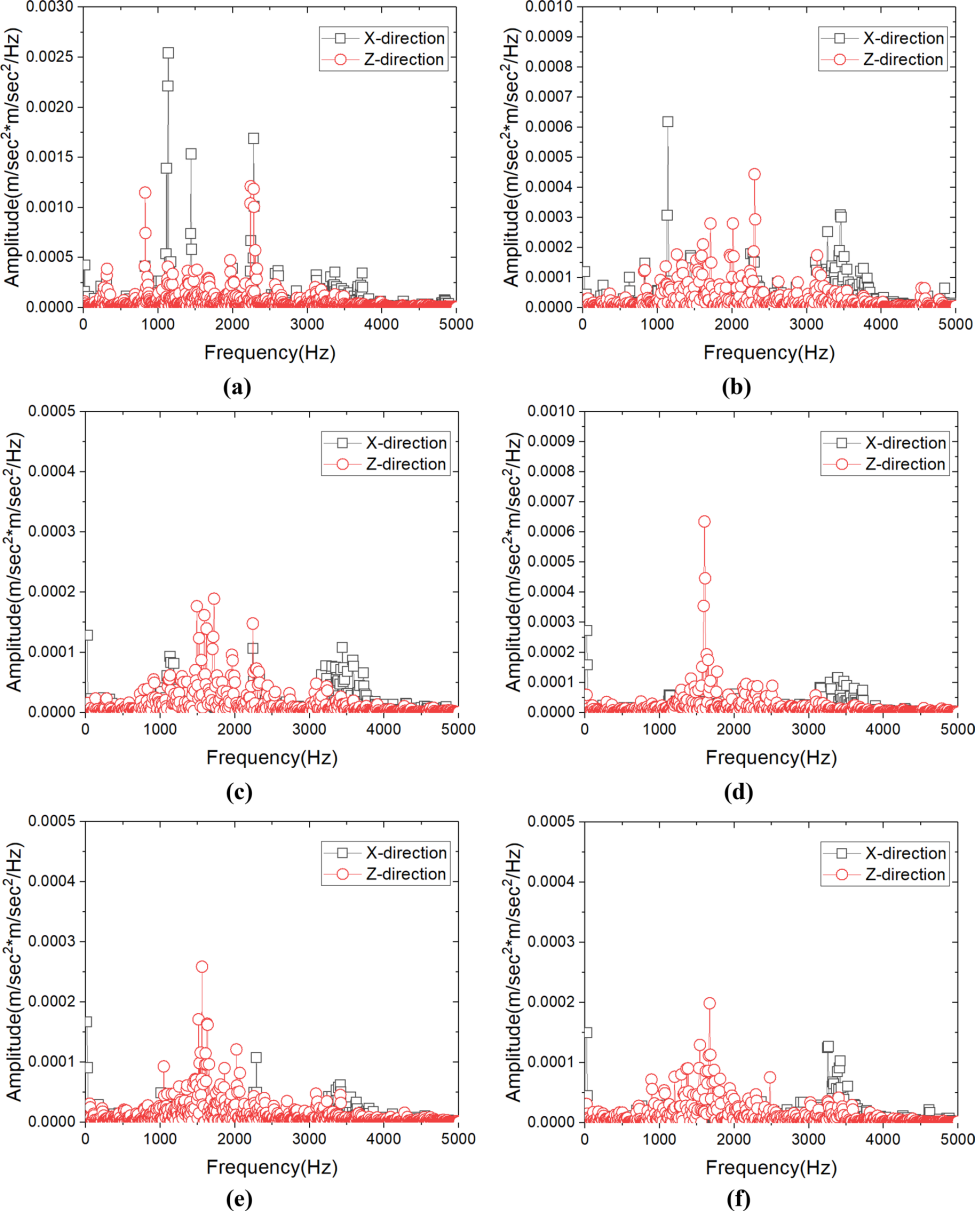
FFT analysis results of the cutting tool vibration signals in X-direction and Z-direction according to undeformed chip thickness; (**a**) undeformed chip thickness 3 μm, (**b**) 2 μm, (**c**) 1 μm, (**d**) 0.5 μm, (**e**) 0.3 μm, (**f**) 0.1 μm.

The measured acceleration signals were converted to frequency domain signal by using fast Fourier transform (FFT) function of using J-beam(Kistler) which was signal processing software. Hamming window was applied to prevent signal leakage of vibration signal with strong aperiodic during FFT. In addition, FFT-based power spectral density (PSD) with little difference depending on the measuring time was applied. Figure 13 shows results that signal of Fig. 12 converted to FFT-based PSD signal. When the undeformed chip thickness was 3 µm, the dominant frequency of the vibration in X-direction was 1135 Hz and this PSD amplitude was 0.0025(m/sec^2^)^2^/Hz as shown in Fig. 13a. In this machining condition, since the undeformed chip thickness was about 6 times thicker than edge radius of cutting tool, the frequency of 1135 Hz can be considered as signal of shearing characteristic. The PSD amplitude of 1135 Hz decrease to 0.0006(m/sec^2^)^2^/Hz at undeformed chip thickness of 2 µm as shown in Fig. 13b, and it converged almost zero below 1 µm as shown in Fig. 13c. This variation of dominant frequency can be seen that shearing characteristic was gradually decreased. On the other hand, when the undeformed chip thickness was reduced to below than 1 µm, the frequency of the vibration signal in Z-direction was concentrated at about 1600 Hz as shown in Fig. 13c, d, and e. It means that the main machining characteristics have been transferred from the X-direction to the Z-direction, because the influence of the plowing and shearing by cutting edge had been increased by size effect. From the above analysis results, the critical DOC may be determined as 1 µm at which the main direction of machining characteristic was converted.

### Machined surfaces according to depth of cut

Above the Sects. 3.1 and 3.2, the critical DOC which occurrence the size effect was determined to by analyzing the force signal and vibration signal. As a result, the variation of the frequency spectrum is effective on the determination of critical DOC more than *K*_*s*_, and it was determined to 1 µm. The effectiveness of the determined critical DOC can be verified by the quality of the machined surface because the plastic deformation was generated by the plowing effect.

**Figure 14:**
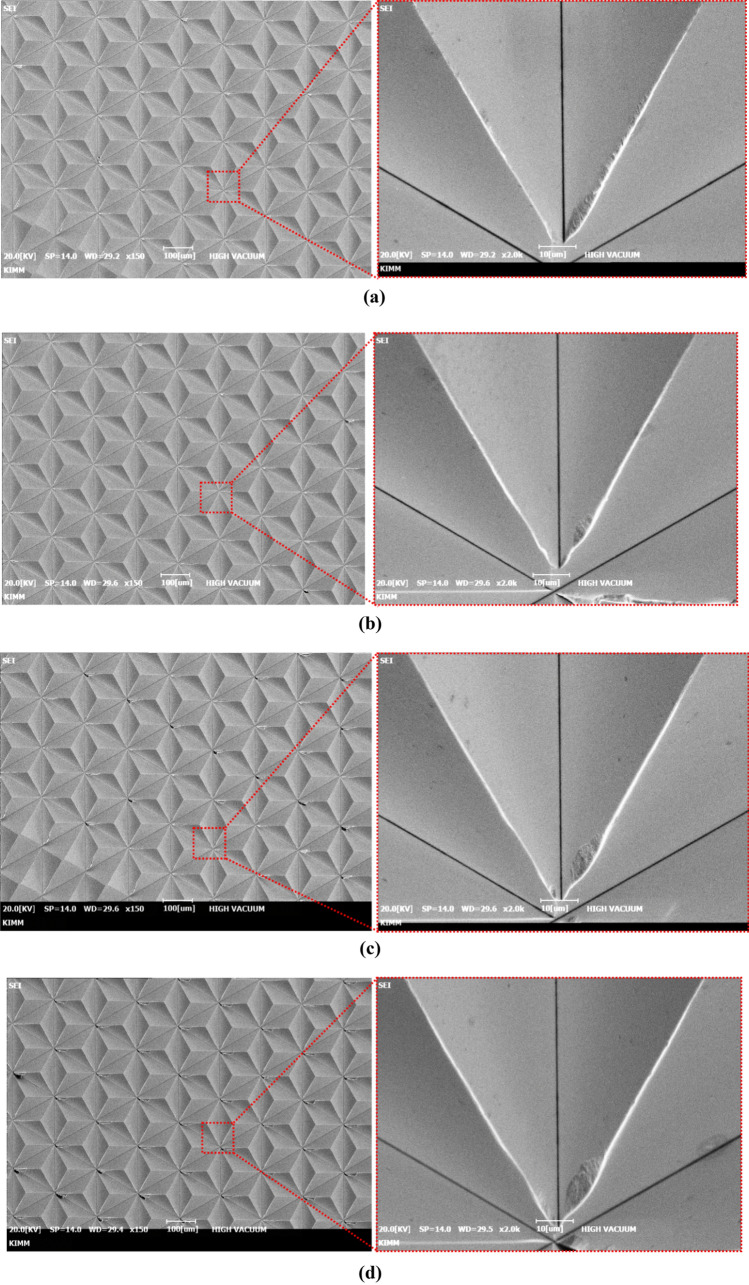

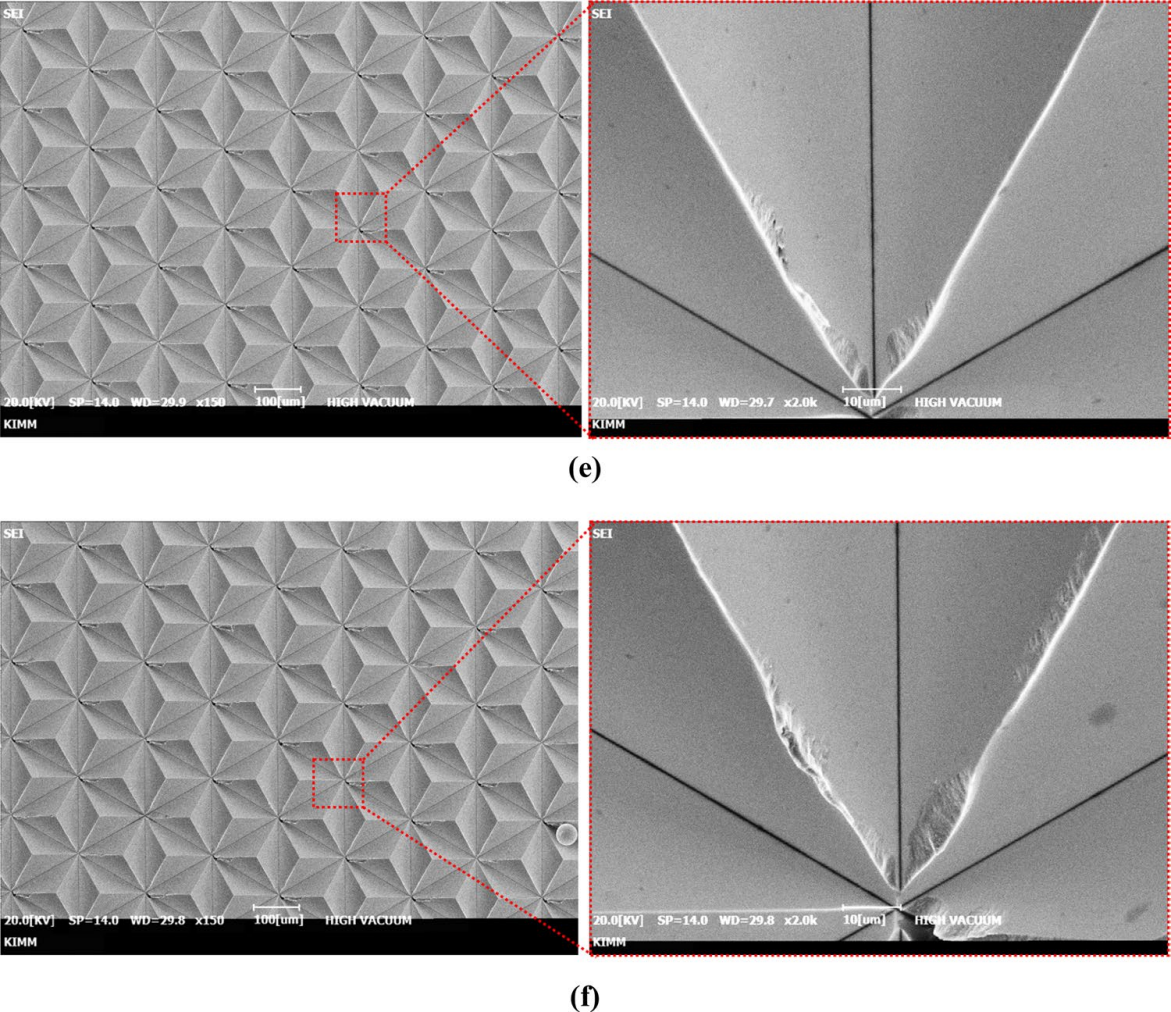
Machined triangular pyramid patterns according to DOC and enlarged image; (**a**) machined surface at the DOC of 3 μm, (**b**) 2 μm, (**c**) 1 μm, (**d**) 0.5 μm, (**e**) 0.3 μm, (**f**) 0.1 μm.

Figure 14a to e show the machined triangular pyramid patterns according to undeformed chip thickness, which was measured by scanning electron microscope. At the DOC of 3 µm, the shape of the exit edge was collapsed due to excessive cutting force, and burrs were formed on the edge of machined pattern as shown in Fig. 14a. The quality of the machined pattern was gradually improved up to critical DOC of 1 µm as shown in Fig. 14c. However, when the DOC was shallower than 1 µm as shown in Fig. 14d, e and f, the edge of the pattern had been blunted due to the plastic deformation affected by plowing effect. In addition, the material fell off from the edge of the pyramid patterns, which intensified the surface damage and increase in the amount of burrs. From these results, the critical DOC was determined to be 1 µm, and this determined value confirmed that same trend as the result of spectral analysis of the vibration signal. Therefore, the frequency spectrum of vibration signal had been verified as an analysis means to determine the optimum depth of cut for machining triangular pyramid pattern with high quality surface and edges. Also, by applying an optimal DOC determined by suggested process, the machined surface and edge quality can be improved, and it can be expected to utilize for mold of advanced optical component required ultra-high brightness retroreflection.

### Fabrication of the master mold and performance test of retroreflection film

**Figure 15:**
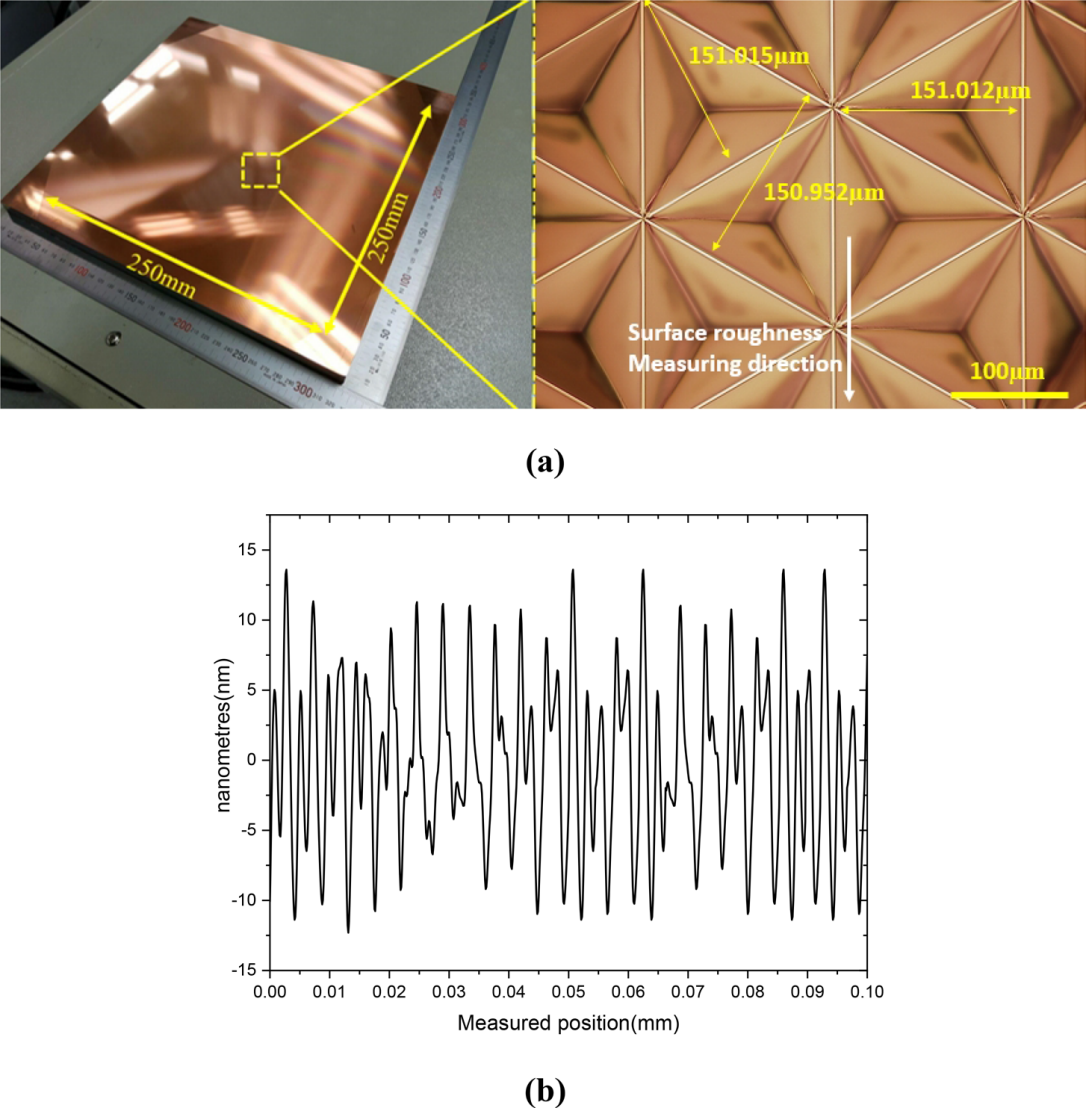
The master mold with area of 250 mm × 250 mm machined by optimized depth of cut (**a**) machined cu-plated master mold with micro triangular pyramid patterns, (**b**) the surface roughness profile of machinerd micro triangular pyramid pattern.

**Figure 16:**
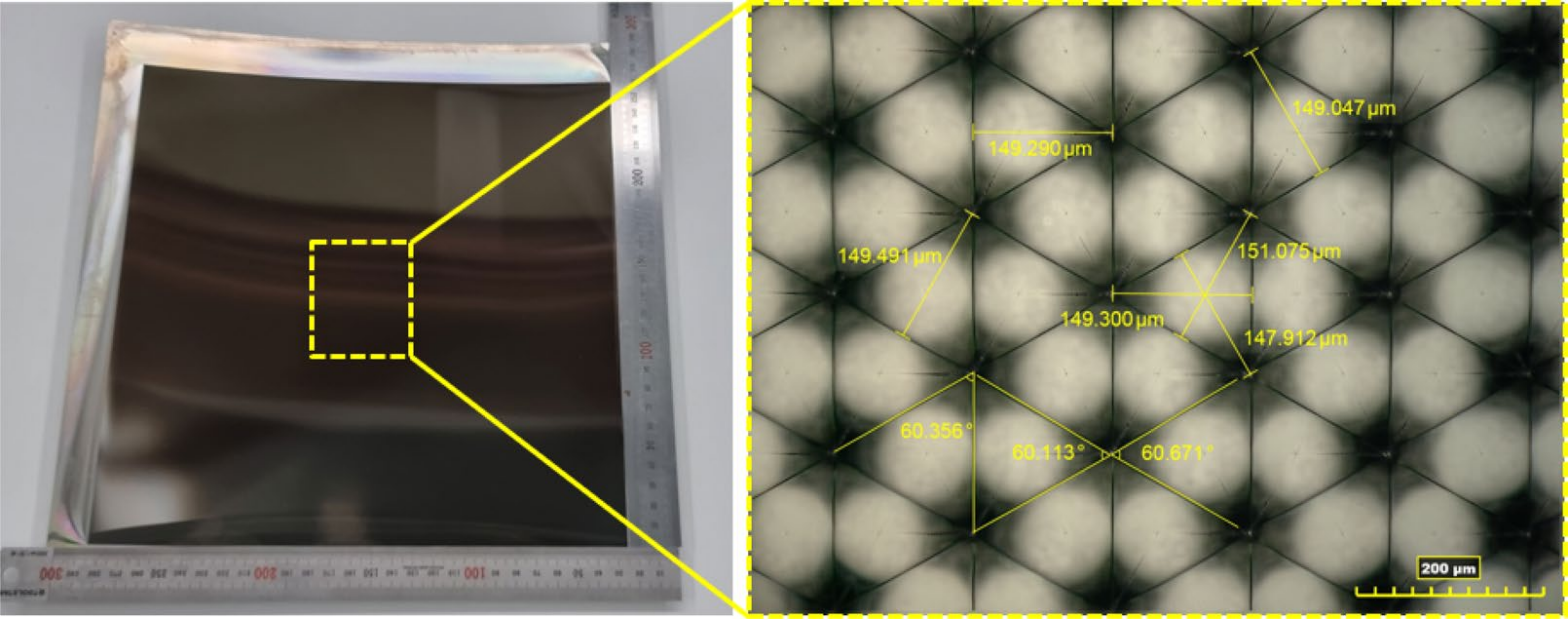
The nickel stamper mold replicated from master mold and enlarged image by optical microscope.

The optimized DOC through the above experimental results was applied to finish process in manufacturing of the master mold with large area of 250 mm by 250 mm. Figure 15 shows the machined master mold, and image measured by 3-D optical microscope(KH-9700, Hirox). The machined triangular pyramid pattern on the cu-plated master mold was machined with high quality of surface roughness 10 nm Ra and high accurate shape under size error of 1 µm. In order to fabricate a high luminance retroreflector, since the retroreflection film has same structures with master mold, the stamper mold having the reverse shape of the master mold using the nickel electroplating replication process was prepared as shown in Fig. 16. The ultra-high luminance retroreflection film was formed using by press molding process.

**Figure 17:**
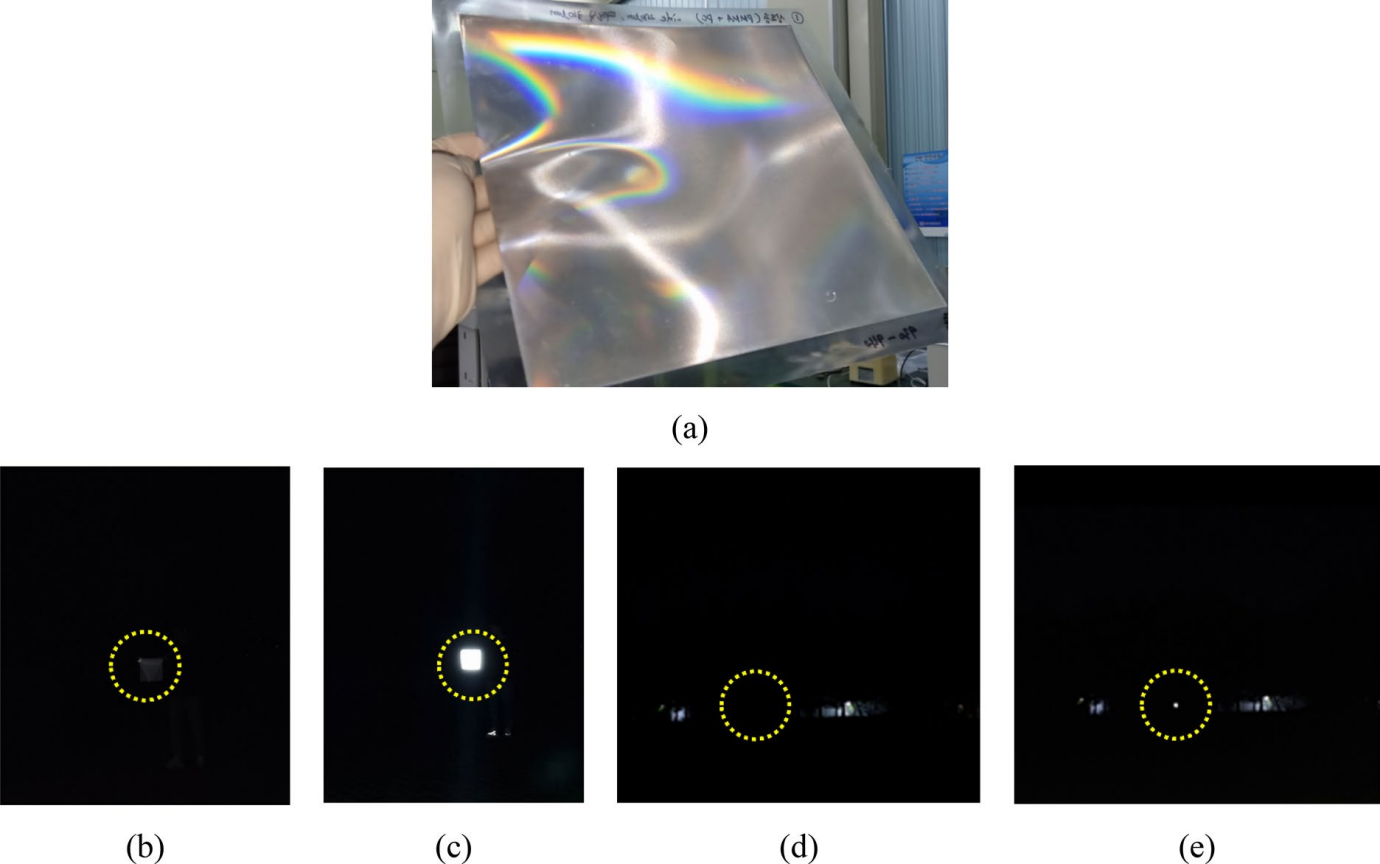
Press molded retroreflection film and performance test (**a**) press molded retroreflection film (**b**) Picture taken without flash from about 10 m away (**c**) Picture taken with flash from 10 m away (**d**) picture taken without flash from about 100 m away (**e**) picture taken with flash from 100 m away (yellow circle indicate the retroreflection film position).

Figure 17a shows the press molded film and the pyramid pattern on this film. The patterns were well formed without size error compared to master mold. However, the edges were little blunt since the two-replication process for fabrication; (i) from master mold to nickel stamper mold, (ii) from nickel stamper mold to retroreflection film. In order to optical performance of retroreflection, photographs were taken in two states which was the flashlight on and off which are with flashlight and without. Pictures are taken by mobile phone camera (Samsung Galaxy S21 ultra) with distance of 10 m and 100 m from retroreflective film. The camera was set to F-number of 1.8, exposure time of 0.1 s, and sensitivity of ISO160. When the picture was taken without flashlight, it is difficult to distinguish the presence of retroreflective film even at a distance of 10 m and 100 m as shown in Fig. 17b and d. However, when the flashlight was on, the retroreflective film can be easily distinguished as shown in Fig. 17c and e. This result was possible because the emitted light was collected by retroreflected to the image sensor without being scattered by the triangular pyramid pattern of the retroreflection film. Also, these results mean the optimization of the critical DOC based on the cutting signal analysis can be utilized for manufacturing master mold of superior high luminance retroreflection film.

## Conclusions

In this paper, determination process for critical DOC was suggested based on analysis of the cutting signals which are cutting force and tool vibration. The signals were converted to cutting resistance and frequency domain. Characteristic of size effect was analyzed by using converted signals, and the frequency variation of the tool vibration was more effective. The optimized critical DOC was applied to fabricate the master mold, and the ultra-high luminance retroreflection film was fabricated. By the considering the above results and discussions, following conclusions can be drawn.

1. In the finish machining of micro triangular pyramid patterns, the RMS value of the cutting force was decreased linearly from 0.3726 N to 0.0567 N according to shallowing the DOC from 3 µm to 0.1 µm. The thrust force has minimum value at the DOC of 2 µm, and the thrusts were higher than cutting force bellow DOC at 500 nm. These variation trend of force signal was assumed by plowing and friction between cutting tool and machining materials.
2. The cutting chip deformation was calculated by using cutting force and thrust force. The deformation ratio of calculated machined chip thickness was significantly increased at DOC of 1 µm, and it was increased to about twice at DOC of 100 nm. The cause of these results were considered by widen shear area of plastic zone and increment of plowing force.
3. The specific cutting energy was calculated using RMS value of the cutting force and cross-sectional cutting area, it shown trends which are slightly increase at the DOC of 1 µm and significant increase below 0.5 µm that increased. For this reason, determination of accurate critical DOC from specific cutting energy was difficult to minimize the cutting energy.
4. From the results of analyzing tool vibration, the dominant frequency in the cutting state was 1135 Hz in X-direction, and the power spectrum of this signal decreased when the DOC was shallow. On the other hand, the power spectrums of tool vibration in Z-direction had higher values than X-direction at 1 µm. From this result, it was considered to occurrence the transition of the main cutting state from the X-direction to the Z-direction, which can be considered as size effect due to the plowing force. Therefore, the critical DOC can be determined more accurately than using the variation of the specific cutting energy.
5. The surface quality of the machined triangular pyramid pattern was deteriorated due to plastic deformation and burrs by size effect at below undeformed chip thickness of 1 µm. From this result, the validity of the determination of the critical DOC using the tool vibration signal was experimentally verified.
6. A high-performance retroreflection film was molded using a press-molding process and a nickel stamper mold, and performance was verified through a simple light reflection test.

This optimizing process was expected to be utilized as a manufacturing process for micro structured mold with high quality surface and accurate edges for advanced optical component with high performance.
